# Study on the mechanism of salt relief and growth promotion of *Enterobacter cloacae* on cotton

**DOI:** 10.1186/s12870-023-04641-w

**Published:** 2023-12-19

**Authors:** Haitao Yue, Shuwen Sun, Ruiqi Wang, Xiaoyun Ma, Shiwei Shen, Yiqian Luo, Xiaoli Ma, Ting Wu, Shuang Li, Zhengyang Yang, Yuxi Gong

**Affiliations:** 1 Laboratory of Synthetic Biology, School of Life Science and Technology, Xinjiang University, Urumqi, 830017 People’s Republic of China; 2 School of Future Technology, Xinjiang University, Urumqi, 830017 People’s Republic of China

**Keywords:** PGPR, Salt relief and growth promotion, Ion uptake, Endogenous hormones, Genome-wide annotation, Multi-omics analysis

## Abstract

**Aims:**

In-depth studies on plant ion uptake and plant growth-promoting rhizobacteria (PGPR) at the molecular level will help to further reveal the effects of PGPR on plants and their interaction mechanisms under salt stress.

**Methods:**

Cotton was inoculated with a PGPR-*Enterobacter cloacae* Rs-35, and the ion uptake capacity, membrane transporter protein activity, and expression of key genes were determined under salt stress. Changes in the endogenous hormone content of cotton were also determined. Further, the genome-wide metabolic pathway annotation of *E. cloacae* Rs-35 and its differential enrichment pathway analysis of multi-omics under salinity environments were performed.

**Results:**

In a pot experiment of saline-alkali soil, *E. cloacae* Rs-35-treated cotton significantly increased its uptake of K^+^ and Ca^2+^ and decreased uptake of Na^+^, elevated the activity of the H^+^-ATPase, and increased the sensitivity of the Na^+^/H^+^ reverse transporter protein on the vesicle membrane. Meanwhile, inoculation with *E. cloacae* Rs-35 could promote cotton to maintain the indole-3-acetic acid (IAA) content under salt stress. Genome-wide annotation showed that *E. cloacae* Rs-35 was respectively annotated to 31, 38, and 130 related genes in osmotic stress, phytohormone and organic acid metabolism, and ion uptake metabolic pathway. Multi-omics differences analysis showed that *E. cloacae* Rs-35 were enriched to tryptophan metabolism, multiple amino acid biosynthesis, carbon and glucose synthesis, and oxidative phosphorylation metabolic pathways at the transcriptome, proteome, and metabolome.

**Conclusion:**

*E. cloacae* Rs-35 can promote cotton balance cell ion concentration, stabilize intracellular IAA changes, stimulate induction of systemic tolerance, and promote the growth of cotton plants under salt stress.

## Introduction

Plant growth-promoting rhizobacteria (PGPR), a large group of bacteria colonized in the plant rhizosphere, can promote plant growth, inhibit plant pathogens and improve the plant rhizosphere environment. Its survival in the plant rhizosphere or surface directly or indirectly promotes or regulates plant growth [1]. Around the year 2000, PGPR-related studies became progressively more widespread. In the process of common adaptation and evolution, PGPR can promote plant growth through various mechanisms. On the one hand, it can directly promote crop growth by producing plant growth regulators, or transform soil minerals through its nitrogen fixation, phosphorus dissolution, and potassium solution, to promote the absorption and utilization of plant-related nutrients [2, 3]. On the other hand, by producing antibacterial substances, inducing crop system resistance, to enhance the resistance of plants [4, 5]. At the same time, PGPR metabolic activities can enhance the decomposition of organic matter in the soil, promote the mineralization of plant nutrient elements, improve the soil structure and nutrient composition, and improve soil fertility [6, 7]. Many reports have researched plant rhizosphere-promoting bacteria in a variety of plant crops, such as tobacco, cotton, and peanut. The results show that PGPR plays an important role in stress resistance, disease prevention, and yield increase of crops [8, 9].

Xinjiang is located in the center of the Eurasian continent. It has a unique climate environment, breeds rich microbial resources, and is an important cotton-producing area in China. Due to the wide area of saline soil in Xinjiang, the development of the cotton planting industry is seriously affected. Excessive accumulation of any form of salt in the soil can adversely affect plant growth and its physiological processes [10]. As one of the main abiotic stresses in nature, salt stress causes osmotic stress, ionic stress, and oxidative stress in plants, hinders the absorption of water and nutrients (roots), and suppresses the normal progress of photosynthesis (leaves). This not only interferes with the normal physiological and metabolic processes in various growth stages, such as plant seed germination, seedling growth, and reproduction but also seriously affects the production potential and function of plants in the ecosystem [11, 12]. Studies have shown that plant–microbe symbiosis can improve plant survival in extreme environments [13, 14], and one way to effectively combat soil salinity is to inoculate plants with PGPR strains to improve salt tolerance [15, 16]. Under salt stress, many PGPR strains reduce the damage of salt stress to plants by changing the selective absorption of Sodium ion (Na^+^), Potassium ion (K^+^), and Calcium ion (Ca^2+^) by plants and maintaining high K^+^/Na^+^ [17, 18]. At the same time, plant hormones participate in the regulation of the whole process of crop growth. These hormones can come from the secretion of plants themselves, called endogenous hormones, and can also come from other sources in vitro, called exogenous hormones. Many microorganisms (some pathogenic bacteria or some growth-promoting bacteria) can affect crops by interfering with plant endogenous hormones or providing exogenous hormones [19]. The changes in different hormone peaks and low differences in cotton plants under salt stress suggest a shift in various physiological processes in cotton. Therefore, it is of great interest to study the PGPR-cotton-salt stress triplet interaction in terms of altered hormone and ion uptake levels.

Meanwhile, with the development of molecular biology technology, multi-group sequencing provides favorable conditions for analyzing the physiological and ecological functions of PGPR, revealing the mechanism of action and metabolic regulation of PGPR [20]. Multi-omics research technology is of great significance for the screening of key genes. By mining relevant functional genes, analyzing the main growth-promoting characteristics of different strains, and deepening the understanding of the life activities of various strains at the molecular level, it can provide theoretical support for strengthening the research of the growth-promoting characteristics of various strains, improving the level of salt stress resistance of plants and the metabolic complementation of strains [21].

At present, many reports have focused on the response of morphological characteristics and physiological indicators of plants after PGPR inoculation. If the study of plant ion absorption level and PGPR molecular level-related gene expression is strengthened, it is beneficial to further reveal the impact of PGPR inoculation on plant salt tolerance and their interaction mechanism under salt stress. *Enterobacter cloacae* Rs-2, Rs-5 and Rs-35 are salinity-promoting bacteria with salinity tolerance that were isolated from the inter-rhizosphere of cotton in salinized soil in Xinjiang in the previous work of our group. The previous experiments investigated the effect of *E. cloacae* Rs-2 and Rs-5 on cotton growth under salt stress [22, 23] and proved that *E. cloacae* Rs-35 have a good salinity-promoting ability with outstanding functions of nitrogen fixation, phosphorus solubilization, and phytohormone secretion [24]. At the same time, *Enterobacteriaceae* is also relatively safe, which has been well developed and utilized in the Aksu region of Xinjiang, and has the value of further research. Therefore, based on the above problems, this paper takes *E. cloacae* Rs-35 as the research object. Firstly, from the three aspects of ion absorption in roots and leaves of cotton plants, the activity of ion transport protein on the membrane, and the expression of key genes of ion partition, we studied and understood the role and mechanism of salt-promoting bacteria *E. cloacae* Rs-35 in ion absorption and balance of cotton. Secondly, from the perspective of changes in endogenous hormone levels, we studied the interaction between PGPR, cotton, and salt stress. Thirdly, to interpret the mechanism of promoting the growth of *E. cloacae* Rs-35 at the molecular level, carry out multi-group annotation and differential analysis, and explore whether there is a metabolic pathway mediated by the genes related to salt detoxification and promoting the growth of *E. cloacae* Rs-35 in the secondary metabolic system. In a word, this article provides a theoretical basis for *E. cloacae* Rs-35 to alleviate salt stress in cotton from the perspectives of ion absorption mechanism, endogenous hormone effect, and annotation of the multi-group metabolic pathway.

## Materials and methods

### Plant growth condition

*Enterobacter cloacae* Rs-35 was cultured in Nutrient agar (NA) liquid medium (beef extract 3 g, peptone 5 g, sodium chloride (NaCl) 5 g, glucose 1 g, agar powder 15 g, 1000ml water, pH7.0–7.2) for 48 h at 200 rpm and 30℃. After shaking and cultivating, centrifuge at 8000 × *g* for 5 min, and then add sterile water. Adjust the optical density (OD) value of the suspension with a spectrophotometer to keep it unchanged.

Cotton seeds (Xinluzao No. 57, which is currently the main cotton variety in the northern Xinjiang cotton region and is purchased from XINJIANG GOLDEN AVENUE SEEDS CO., LTD.) were sterilized with 70% alcohol for 5 min, soaked in 0.1% mercuric chloride solution for 10 min, washed in sterile distilled water for 3 times, and then soaked in *E. cloacae* Rs-35 bacterial suspension for 4 h as the treatment group (RS35), and soaked in sterilized distilled water as the control group (CK). The cotton seeds of the control group and the treatment group grow in a flowerpot with sterile soil, and the light intensity is 280 μmol·m^−2^·s^−1^, humidity 40%, and temperature 28℃ in the light incubator. The control group and the treatment group were irrigated with Hoagland nutrient solution (NS) and Hoagland nutrient solution containing 0.8% NaCl (S), and 50 mL bacterial solution was added to each bacterial suspension treatment group on the third day. After the main leaves of cotton grew out, we began to collect and measure the follow-up samples of each experimental group, and each parallel experiment was repeated 3 times.

### Determination of the ion concentration in cotton

Regularly observe the emergence of seedlings, and measure the element absorption when the long cotton grows to two leaves and one heart. Select the cotton plants with the same growth in each experimental group, sample the cotton roots and leaves separately, wash them with distilled water, absorb them, kill them at 110℃ for 5 min, and then dry them at 80℃ to constant weight, grind them, and pass a 1 mm sieve. Accurately weigh 0.1 g of dry sample and put it into a 100 ml digestion tube, add 5 ml of concentrated H_2_SO_4_, shake it well, heat it for digestion, take it off when the solution is brownish black, add a few drops of H_2_O_2_ after slightly cooling, and then heat it to slightly boiling, and then digest it for about 7–10 min, continue to add H_2_O_2_ after slightly cooling, and continue to digest it, and repeat this for several times, until the solution is colorless or clear, take it off and cool it, and put it into a 100 ml volumetric flask. Suck the supernatant to determine nitrogen (N) (Kjeldahl nitrogen determination method), phosphorus (P) (molybdenum-antimony colorimetry), potassium (K), and sodium (Na) (AA240 atomic absorption emission spectrometer, Agilent) respectively. Repeat three times for each sample, and finally obtain the content of five elements Na, calcium (Ca), K, cutter (Fe), and magnesium (Mg) in cotton samples.

#### Membrane microcapsule inorganic phosphorus and protein content determination and membrane proton pump activity calculation

Regularly observe the emergence of seedlings, and collect samples of the cotton plasma membrane and vacuolar membrane roots and leaf microcapsules after the long cotton grows true leaves. Weigh 2g of young roots or leaves of 2cm segment of the cotton root tip, add 6ml of grinding fluid (25mmol/L Hepes-Tris (PH7.4), 250 mmol/L mannitol, 125 mmol/L KCl, 2 mmol/L ethylene glycol tetraacetic acid (EGTA), 10 g/L, 2 mmol/L phenylmethyl sulfonyl fluoride (PMSF), 5 mmol/L ethylene diamine tetraacetate acid (EDTA), 10 g/L polyvinylpolypyrrolidone (PVPP), 1 mmol/L dithiothreitol (DTT), 1 g/L bovine serum albumin (BSA) (the latter two are added before use)), and grind on the ice bath. The obtained homogenate is filtered with 2 layers of sterile gauze, and the filtrate is 500 × *g* centrifuge for 10min and take the supernatant with 10000 × *g* centrifuge for 15min, and then take the supernatant with 60000 × *g* centrifuge for 30 min, discard the supernatant, and precipitate with 1 ~ 2 mL suspension (25 mmol/L Hepes-Tris (PH7.4), 250 mmol/L mannitol, 1 mmol/L DTT, 300 mmol/L sucrose, 1 mmol/L EGTA, 125 mmol/L KCl, ready for use) for suspension. Carefully spread the suspended homogenate onto the discontinuous sucrose gradient solution (the concentration from top to bottom is 220g/L, 360g/L, 450g/L), after 70000 × *g* centrifugation for 2 h, slowly collect the part between 220 ~ 360 and 360 ~ 450 with a pointed pipette. They are vacuolar membrane microcapsule preparation and plasma membrane microcapsule preparation respectively. Place the preparation in a 1.5 mL Eppendorf tube and store it in an ultra-low temperature refrigerator (-80℃) for use. (All reagents and operations are carried out on ice).

0.5 mL of reaction solution contains 150 μl 100 mmol/L Hepes-Tris (PH7.5), 50 μl 20 mmol/L MgSO_4_, 50 μl 500 mmol/L KCl, 50 μl 5 mol/L NaN_3_ (inhibition of mitochondrial activity), 50 μl 1 mmol/L ammonium molybdate (inhibiting non-specific phosphatase activity), 50 μl 1 mmol/L sodium vanadate (inhibiting ATPase activity of plasma membrane), 50 μl film microcapsule preparation, with 50 μl 20 mmol/L ATP-Tris (PH7.5), starts the reaction. Put the reaction tube in a warm water bath at 37℃, react for 20 min, add 1 mL of reaction termination solution (5% ammonium molybdate, 5 mol/L H_2_SO_4_, mixed with ammonium molybdate: H_2_SO_4_: H_2_O = 1:1:3), and then add 0.2 mL of color solution (0.25 g of aminophenol sulfonic acid is dissolved in 100 mL of 1.5% Na_2_SO_3_ solution, PH is adjusted to 5.5, and then add 0.5 g of Na_2_SO_4_ to dissolve, and mix well), shake well, place at room temperature for 40 min, and then conduct spectrophotometer colorimetry at 660 nm. The blank control was those who added the termination solution before the reaction. Prepare 0, 2, 4, 6, 8, and 10 μmol/L of KH_2_PO_4_ standard solution, same as the method above, take it 50 μl respectively to replace the membrane microcapsule preparation and add it into the reaction system for reaction, then conduct spectrophotometer colorimetry at 660 nm, and finally obtain the inorganic phosphorus standard curve.

Bradford method was used for protein quantification, and BSA was used as the standard protein. 50 μl of membrane microcapsule preparation was added to the reaction tube, and then 5 mL of protein reagent (containing 0.01% (w/v) Coomassie Brilliant Blue G-250, 4.7% (w/v) ethanol, and 8.5% (w/v) phosphoric acid; filtered them before use) was added to react for 5 min, and then colorimetric at 595 nm was performed within 2 ~ 60 min. Prepare gradient concentrations of 0, 0.2, 0.4, 0.6, 0.8, and 1.0 mg/mL with BSA, separately taking 50 μl of it was added to 5 ml of protein reagent, reacted for 5 min, and compared at 595 nm in 2 ~ 60 min to obtain the protein standard curve.

Calculate the inorganic phosphorus content and protein content according to the standard curve, calculate the H^+^-ATPase activity in combination with the reaction time (20 min), and use μmolPi/ (mgprotein × h) represents.

#### Analysis of *GhNHX1* gene expression by real-time PCR

The total RNA of cotton leaves and roots was extracted and reversely transcribed into cDNA. The Na^+^/H^+^ reverse transport protein coding gene (*GhNHX1*) and housekeeping gene (*cdn1*, encoding the juniper synthetase) on the vacuolar membrane were amplified by polymerase chain reaction (PCR). The PCR products were ligated with the cloning vector *pMD19-T* respectively, to construct the recombinant plasmids *pMD19-T*/*GhNHX1* and *pMD19-T*/*cdn1*, and verified by digestion with BamH I and Hind III. The primers for Real-Time PCR of *GhNHX1* and *cdn1* were designed (Table 1), and the quantitative standard curves of *GhNHX1* and *cdn1* were drawn with standard quality particles, then the Real-Time PCR amplification detection of the two genes (SYBR Green method) was performed. According to the double standard curves of housekeeper gene *cdn1* and target gene *GhNHX1*, the system automatically gave the quantitative results of *cdn1* and *GhNHX1* in each sample. According to these two quantitative values, the quantitative results of *cdn1* are removed with the quantitative results of the target gene *GhNHX1*, and the final correction value is obtained. To make it easier to compare the expression amount between samples, take the lowest expression amount of the control sample (CK-Leaf sample at the 48th hour, the data is not given here), and then take the logarithm with the bottom of 10 to finally get the relative expression amount of *GhNHX1* gene.

**Table 1:** Primers of *GhNHX1* and *Cdn1*

Genes	GenBank ID	Primer sequence		Length
*GhNHX1*	AF515632	Forward	5’-CAATCCAGAGTTTTGACCTCGTT-3’	121bp
		Reverse	5’-CCAACAATCACTCCCAGCATAG-3’	
*Cdn1*	AF27042511	Forward	5’-AGTCCAAAGACTGGGTGTGAGTT -3’	114bp
		Reverse	5’-CGAAGGGATGTGGTGTAGAGG -3’	

### Determination of endogenous abscisic acid content in cotton

Cotton samples were collected from each treatment after every 24 h. Each time, 5 g of fresh cotton tissue was taken (accurately weighed), and immediately frozen and ground with liquid nitrogen. 30 ml of 80% methanol solution was added to homogenize for 5 min, and then vibrated for 24 h at 4℃, and filtered. The residue is dissolved in 20 ml of 80% methanol and shaken for 1 h, repeated twice, and the filtrate is combined. The filtrate is dried and evaporated (36℃-38℃) to 1/2 of the original volume on the rotary evaporator. Add 10 ml of petroleum ether to extract the pigment, take out the lower methanol solution (discard the petroleum ether layer), and continue to decompress and concentrate to the aqueous solution. Use 1 mol/L HCl to adjust the pH of the aqueous solution to 2.6, and then extract it with an equal volume of ethyl acetate. Collect the ethyl acetate phase, extract the aqueous phase with ethyl acetate twice, and finally merge the ethyl acetate phase. Concentrate and evaporate dry with a rotary evaporator. Dissolve the residue with 4 ml of 100% methanol solution and take out 200 μl of the obtained solution is evaporated to methanol under 40℃, and 0.1 ml of N, O-bis-trimethylsilyl acetamide (BSA2) is added for silane reaction for 30 min, and 1 μl was analyzed by gas chromatography (GC) and quantified by external standard method. The GC system is SHIMADZU GC 2010 Gas Chromatography with FID detector. Gas chromatography was performed on a DB-1 column (Bio-Rid). Injection temperature: 270 °C, injection mode: split flow; carrier gas: N_2_; control mode: linear speed; pressure: 95.7Kpa; total flow rate: 21.7 ml/min; column flow rate: 0.6 mL/min; linear speed: 22.8 cm/s; purge: 3.0 mL/min; split ratio: 30.7; column oven: 240℃; equilibrium time: 3 min; detector: FID 270℃; stop time: 5 min; makeup: 40 mL/min; H_2_: 40.0 mL/min; air: 400.0 mL /min. Use 100% methanol solution to prepare abscisic acid (ABA) standard with concentrations of 0 μg/mL, 25 μg/mL, 50 μg/mL, 75 μg/mL, 100 μg/mL, 125 μg/mL, 150 μg/mL, 175 μg/mL, 200 μg/mL solution, 0.1 ml of BSA2 was added respectively for 30 min of sialylation reaction, and extract 1 μl for GC analysis. The standard curve is made according to the peak area, and the regression equation of peak area and ABA concentration is obtained. The peak area of the sample is substituted into the equation to obtain the amount of ABA produced by the sample. Calculate the amount of ABA per gram of sample. The difference in ABA production of cotton plants with different treatments was analyzed.

### Determination of endogenous indole-3-acetic acid content in cotton

Collect cotton samples from each treatment every 24 h, take 5 g of fresh cotton tissue each time, freeze and grind it with liquid nitrogen immediately, add 80% methanol aqueous solution to 10 times the volume of plant tissue for homogenization, and react the homogenization at -15℃ for 90 min, wash the residue with 10 ml of 80% methanol after filtration, and collect the filtrate. The methanol is evaporated by rotary evaporation at 40℃. The residue is dissolved with 15 ml of 0.5 mol/L phosphoric acid buffer solution and finally filtered to precipitate. The filtrate was extracted twice with ether, the ether phase was retained, the water phase was adjusted to PH = 3 with 3 mol/L phosphoric acid, and then extracted twice with ether, and the ether phase was combined. Extract twice with 10 ml 0.05 mol/L phosphoric acid buffer, combine the phosphoric acid buffer phase, adjust the PH to 3 with 0.3 mol/L phosphoric acid, and extract once with 20 ml ether. The ether is evaporated by rotary evaporation, and the dry matter is dissolved in 2 ml of methanol. After centrifugation of the obtained solution, take the 180 μl of supernatant, add 1320 μl (PH = 7.2) of phosphoric acid buffer, and 1500 μl reagent R1 reagent (volume ratio = 12 g/L FeCl_3_:7.9 mol/L H_2_SO_4_ = 1:49). React at room temperature for 20 min, and then conduct spectrophotometric analysis at the wavelength of 535 nm, and use an external standard method for quantification. Use standard indole-3-acetic acid (IAA) and phosphate buffer with PH = 7.2 to prepare respectively the concentration of 5 μmol/L, 10 μmol/L, 20 μmol/L, 40 μmol/L, 60 μmol/L, 80 μmol/L of IAA solution, under the same conditions, carry out the spectrophotometric analysis at the wavelength of 535 nm, draw the standard curve according to its absorbance, and obtain the regression equation of absorbance and IAA concentration. Substitute the peak area of the sample into the equation, and obtain the amount of IAA produced by the sample. Calculate the amount of IAA per gram of sample.

### Whole-genome sequencing and metabolic pathway analysis

Inoculate saline-alkali resistant strain *E. cloacae* Rs-35 with 3% (v/v) inoculum in Luria–Bertani (LB) liquid medium, culture at 37℃ and 150 rpm/min for 10 h to the exponential growth stage, then centrifuge at 4℃ and 5000 × *g* for 5 min, and immediately freeze 10 min at -80℃ in liquid nitrogen, store in refrigerator at -80℃ until dry ice sample delivery. The whole genome of the *E. cloacae* Rs-35 strain was sequenced based on the Illumina NovaSeq sequencing platform. The complete sequence is obtained after the sequencing data is filtered and sorted. The obtained gene sequence and Rapid Annotation Using Subsystem Technology (RAST, https://rast.nmpdr.org/) platform are used to analyze the metabolic pathway of *E. cloacae* Rs-35, and the whole genome data of *E. cloacae* Rs-35 strain is deeply excavated, and the genes and related metabolic pathways related to the main growth promotion performance in the genome of *E. cloacae* Rs-35 strain are studied. The sequencing work is entrusted to Shanghai Meiji Biotechnology Co., Ltd.

#### Response of strain *E. cloacae* Rs-35 to the saline environment at the transcription, translation, and metabolic levels

Inoculate saline-alkali resistant strain *E. cloacae* Rs-35 with the ability of salt solubilization and growth promotion in LB liquid medium with 3% (v/v) inoculation amount, and cultured at 37℃, 150 rpm/min for 10 h until the exponential growth period, and the seed liquid was obtained. Inoculate with 3% (v/v) inoculum in normal conditions (PH = 7.0, NaCl = 10 g/L) LB liquid medium and high saline-alkali (PH = 10.0, NaCl = 60 g/L) LB liquid medium, respectively, at 37℃ and 150 rpm/min. After culturing to the index stage, take 50 mL of bacterial solution respectively and transfer it to 50 mL sterile centrifuge tube, centrifuge for 5 min at 4℃ and 5000 × *g*, and remove the supernatant. Rinse the obtained bacteria with enough sterile water, centrifuge for 5 min at 4℃ and 5000 × *g*, remove the supernatant, continue to wash with sterile water, and repeat three times. The obtained cells were frozen with liquid nitrogen for 10 min and then placed at -80℃ until the sample was sent by dry ice. Three biological replicates were made for each treatment, and the transcriptome, proteome, and metabolome under the two treatment conditions were prepared for testing. Send it to Novogene Co., Ltd for transcriptome and proteome sequencing analysis, and send it to BGI Genomics Co., Ltd for metabolome sequencing analysis. After the data were released, the bioinformatics method was used to analyze the metabolic pathway annotation of the differential expression genes and the differential metabolites of *E. cloacae* Rs-35 strain in two environments, explore the response mode of *E. cloacae* Rs-35 strain to the saline-alkali environment at the transcription level, translation level, and metabolic level, and further study the growth promotion mechanism of *E. cloacae* Rs-35 strain in alleviating cotton salt stress.

### Statistical analysis

Statistical analysis was carried out with the R software. The non-parametrical Mann–Whitney test was used for the different comparisons of means. Means that were not significantly different (*P* > 0.05) show the same letter in graph representations.

## Results

### Effect of *Enterobacter cloacae* Rs-35 on ion absorption of cotton under salt stress

**Fig. 1:**
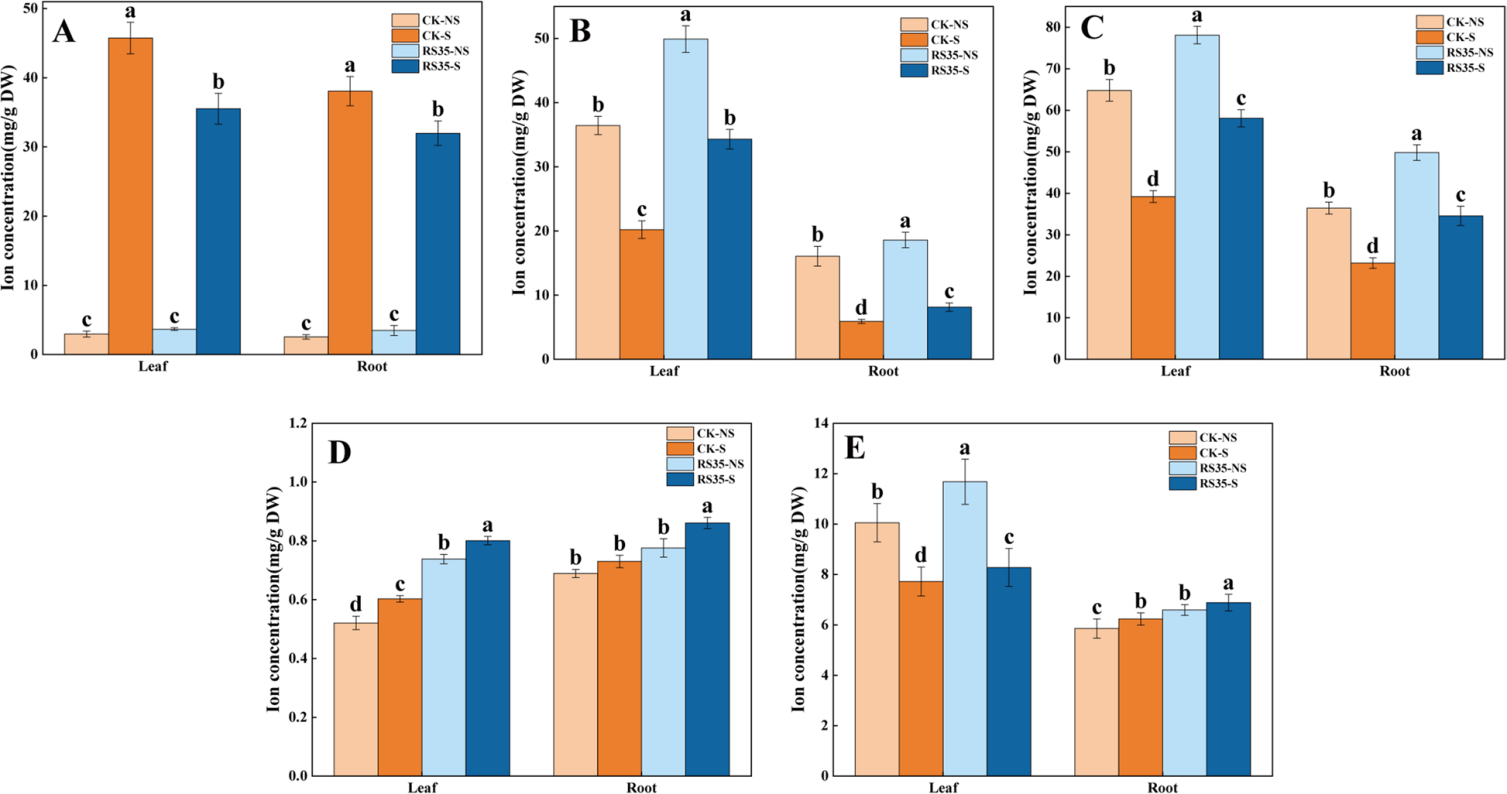
Effects of sodium chloride (NaCl) stress and Enterobacter cloacae Rs-35 on leaf and root ion absorption in cotton. **A** Na. **B** Ca. **C** K. **D** Fe. **E** Mg. Data are presented as the mean ± SD (*n* = 3). Different letters denote significant differences between treatments (Mann–Whitney test, *P* < 0.05)

The concentrations of sodium (Na), calcium (Ca), potassium (K), iron (Fe), and magnesium (Mg) were determined in the leaves and roots of cotton, showing that salt stress had different effects on the absorption of the five ions in cotton (Fig. 1). In the salt environment (S), the Na content in cotton leaves and roots increased significantly, Ca and K content decreased significantly, Fe content increased slightly, and there was no significant consistent variability in Mg content. Compared to the non-salt treatment (NS), the ratio of K/Na and Ca/Na in salt treatment decreased, showing that salt stress affected the ion balance in cotton plants. However, it is worth noting that compared with the control group (CK), in the experimental group (RS35) treated with *E. cloacae* Rs-35, the content of K and Ca significantly increased, the content of Na significantly decreased, and the content of Fe and Mg slightly increased in the salt environment. The results show that *E. cloacae* Rs-35 promotes the absorption of K and Ca and regulates the ion ratio of K/Na and Ca/Na, reduces the environmental stress of cotton growth, and maintains the ion balance of cotton plants.

### Effect of *E. cloacae* Rs-35 on the activity of cotton membrane proton pump H^+^- ATPase

**Fig. 2:**
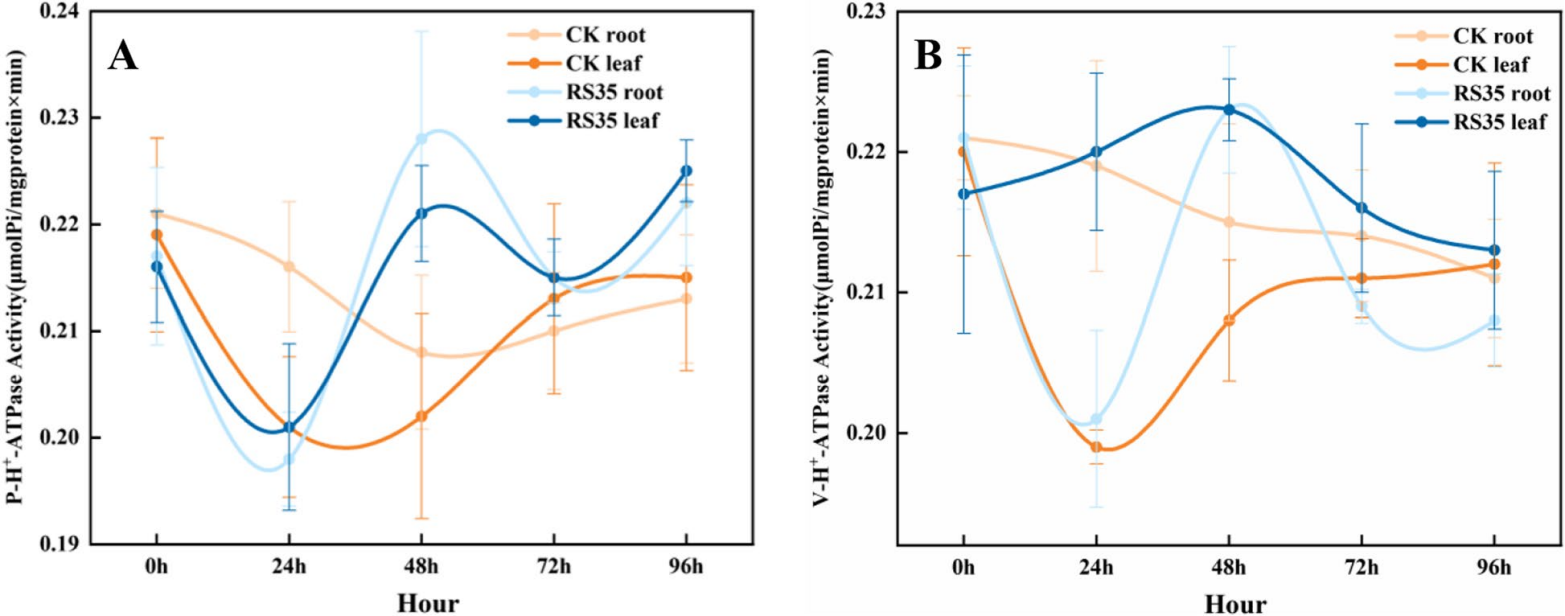
Effects of *E. cloacae* Rs-35 on the proton pump activity of the cotton membrane. **A** Plasma membrane. **B** Vacuole membrane. Data are presented as the mean ± SD (*n* = 3)

The changes in proton pump activity of the plasma membrane (P–H^+^-ATPase) of cotton roots and leaves under the stress of 0.8% sodium chloride (NaCl) are shown in Fig. 2A. Under non-salt stress (0 h), the ATPase activity of root and leaf plasmalemma in the control group was equivalent. At the 24th hour of NaCl stress, the ATPase activity of leaf plasmalemma decreased rapidly and began to rise rapidly at the 48th hour. At the 96th hour, the ATPase activity of leaf plasmalemma rose to a slightly lower level than that without salt. The plasma membrane ATPase activity of roots began to decline slowly after salt stress and was consistent with that of leaves at 96 h. In contrast, the ATPase activity of cotton roots inoculated with *E. cloacae* Rs-35 group reached the lowest value and the peak value at the 24th and 48th hours, respectively, and changed sharply, and tended to be stable after 72 h, and the activity was slightly lower than the control at the same time. The activity of ATPase in leaves rose steadily and reached the peak at 48 h, then decreased slowly. At 96 h, the activity was slightly higher than the control at the same time. Overall, the ATP enzyme activity changes of the cotton root and leaf plasma membrane were generally decreased under NaCl stress, and the leaf plasma membrane activity rebounded after 24 h. The plant growth promoting rhizobacteria (PGPR) *E. cloacae* Rs-35 has an impact on the activity of cotton plasma membrane ATPase, which makes the sensitivity of cotton plasma membrane ATPase activity to salt stress increased, and the activity changes in a wide range, especially in the root plasma membrane ATPase activity.

Under the stress of 0.8% NaCl, the changes in the activity of the vacuolar membrane proton pump (V-H^+^-ATPase) in cotton roots and leaves are shown in Fig. 2B. Before salt stress (0 h), the vacuolar membrane ATPase activity of roots and leaves in the control group was the same. After NaCl stress, the vacuolar membrane ATPase activity of leaves and roots rapidly decreased to the lowest value and rose slowly after 48 h. At 72 h, the vacuolar membrane ATPase activity of leaves exceeded the vacuolar membrane ATPase activity of roots, and at 96 h, both of them recovered slightly lower than the level of activity without salt. In contrast, under the influence of the *E. cloacae* Rs-35 strain, the ATPase activity of cotton roots and leaf vacuole membrane showed the same trend, and the activity was not different, which decreased significantly at the 24 h, increased significantly at the 48 h, and decreased at the 72 h, showing staggered fluctuations. Notably, the ATPase activity of both cotton roots and leaf vacuoles was higher after 36 h compared with the concurrent control. In general, under NaCl stress, the activity of vacuolar membrane ATPase in cotton roots and leaves generally showed a trend of decreasing first and then increasing, and the salt solubilizing bacteria *E. cloacae* Rs-35 had an impact on the activity of cotton vacuolar membrane ATPase, significantly increased the activity of cotton roots and leaves vacuolar membrane ATPase, enhanced its sensitivity to salt stress, and the range of activity change was large.

### Detection of the effect of *E. cloacae* Rs-35 on the expression of GhNHX1 by Real-Time PCR

The expression of the Na^+^/H^+^ reverse transport protein coding gene (*GhNHX1*) on the vacuolar membrane was analyzed by the Real-Time PCR method. The results of the fusion curve analysis of the reaction amplification fragments of *GhNHX1* and *Cdn1* showed that the two reaction fusion curves were single peaks and the peaks were single, which showed that the products of the two amplification reactions were specific, and the two pairs of primers used had strong specificity.

**Fig. 3:**
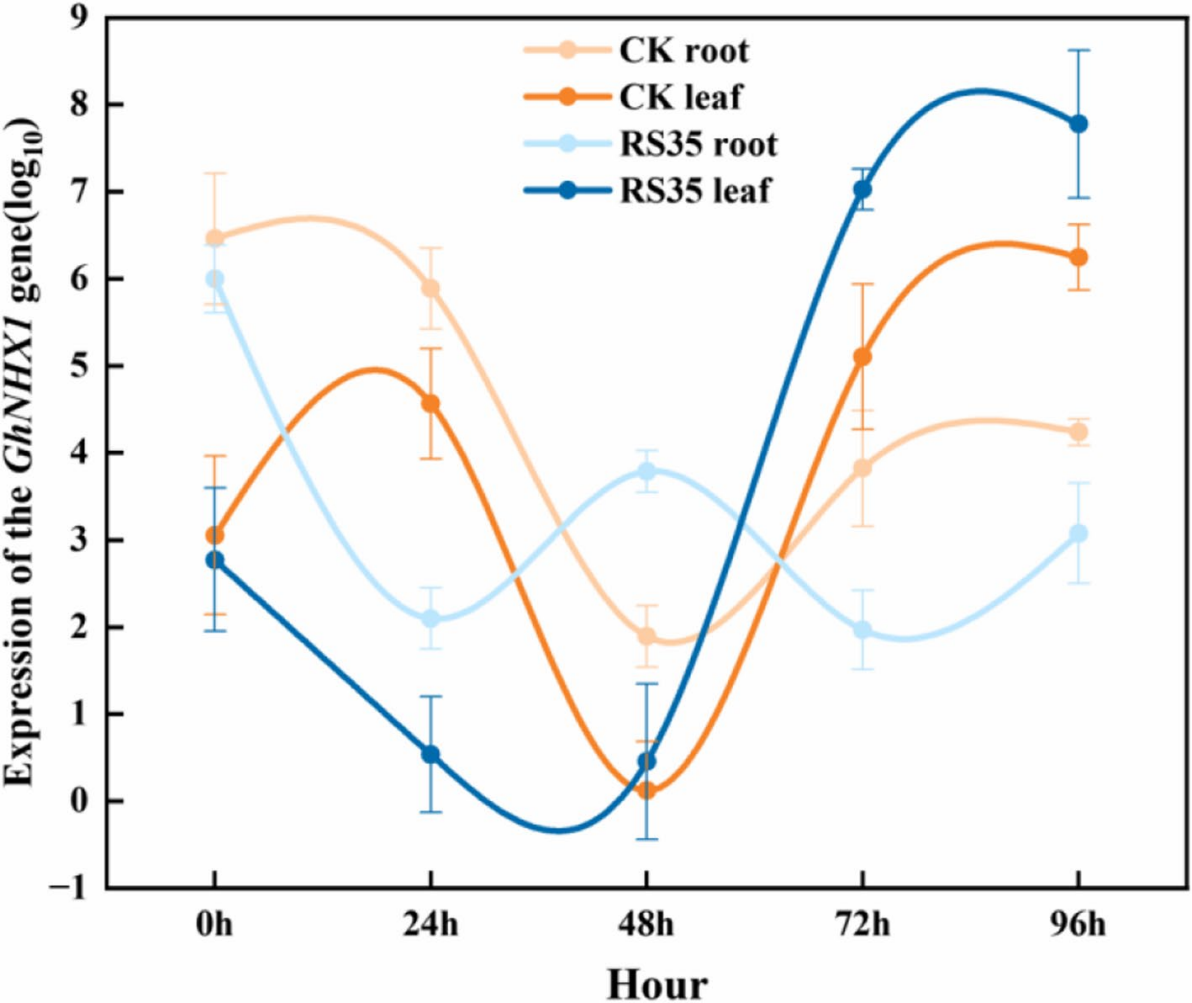
Effect of *E. cloacae* Rs-35 on vacuolar GhNHX1 gene expression in cotton roots and leaves. Data are presented as the mean ± SD (*n* = 3)

The temporal expression change of cotton *GhNHX1* under 0.8% NaCl stress is shown in Fig. 3. Before salt stress (0 h), the expression of *GhNHX1* in CK roots was higher than that in leaves. After NaCl stress was added, the expression of *GhNHX1* in leaves and roots decreased rapidly, reached the lowest point at 48 h, and then began to rise. At 96 h, the expression of *GhNHX1* in leaves had far exceeded the expression at 0 h, but the expression of *GhNHX1* in roots had not recovered to the level at 0 h. The expression of *GhNHX1* in roots and leaves showed a trend of decreasing first and then increasing, indicating that NaCl stress stimulated the expression of *GhNHX1* in leaves and inhibited the expression of the *GhNHX1* gene in roots. The initial value of *GhNHX1* expression in cotton leaves and roots of the *E. cloacae* Rs-35 treatment group was slightly lower than that of the control group and began to decline after salt stress. The expression of *GhNHX1* in leaves began to increase after 48 h, and was always higher than that of the control group; The expression of *GhNHX1* in the roots began to increase after 72 h, which did not exceed the expression of *GhNHX1* in the roots of the control group, indicating that the inoculation of salt solubilizing bacteria *E. cloacae* Rs-35 played a more useful role in alleviating salt stress on the physiological metabolism of cotton leaves.

### Effects of *E. cloacae* Rs-35 on endogenous hormones of cotton

**Fig. 4:**
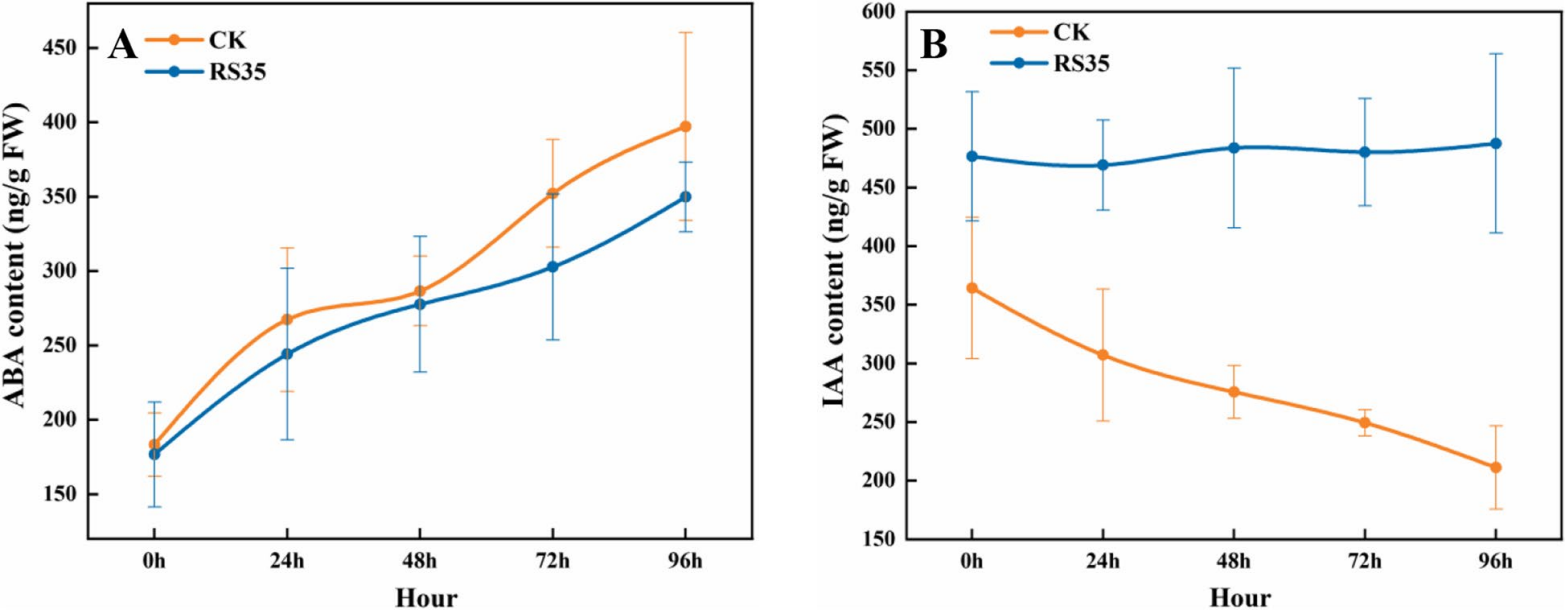
Changes of ABA (**A**) and IAA (**B**) contents of cotton. Data are presented as the mean ± SD (*n* = 3)

The endogenous hormones abscisic acid (ABA) and indole-3-acetic acid (IAA) in cotton were determined, and the results are shown in Fig. 4. It can be seen that under salt stress, the ABA content of *E. cloacae* Rs-35 treatment and control cotton plants showed an obvious upward trend. The content of ABA in the control doubled from 190.65 ng/gFW to 391.37 ng/gFW; The content of ABA in the treated cotton plants also basically doubled. Unlike the control, the initial content of ABA in the treated cotton plants was lower, 183.78 ng/gFW. At the same time, salt stress will cause the IAA content of cotton plants to decrease. The IAA content of control cotton plants decreased by 41.5% after 96 h, but the IAA content of cotton plants inoculated with *E. cloacae* Rs-35 remained unchanged (decreased by 3.8%). It can be concluded that *E. cloacae* Rs-35 treatment will have an impact on the content of endogenous hormones in cotton under salt stress, with little impact on the content of ABA, but a significant impact on the content of IAA, so that it can maintain a relatively stable content under salt stress.

### *E. cloacae* Rs-35 genome-wide annotation and analysis of genes related to salt relief and growth promotion

**Fig. 5:**
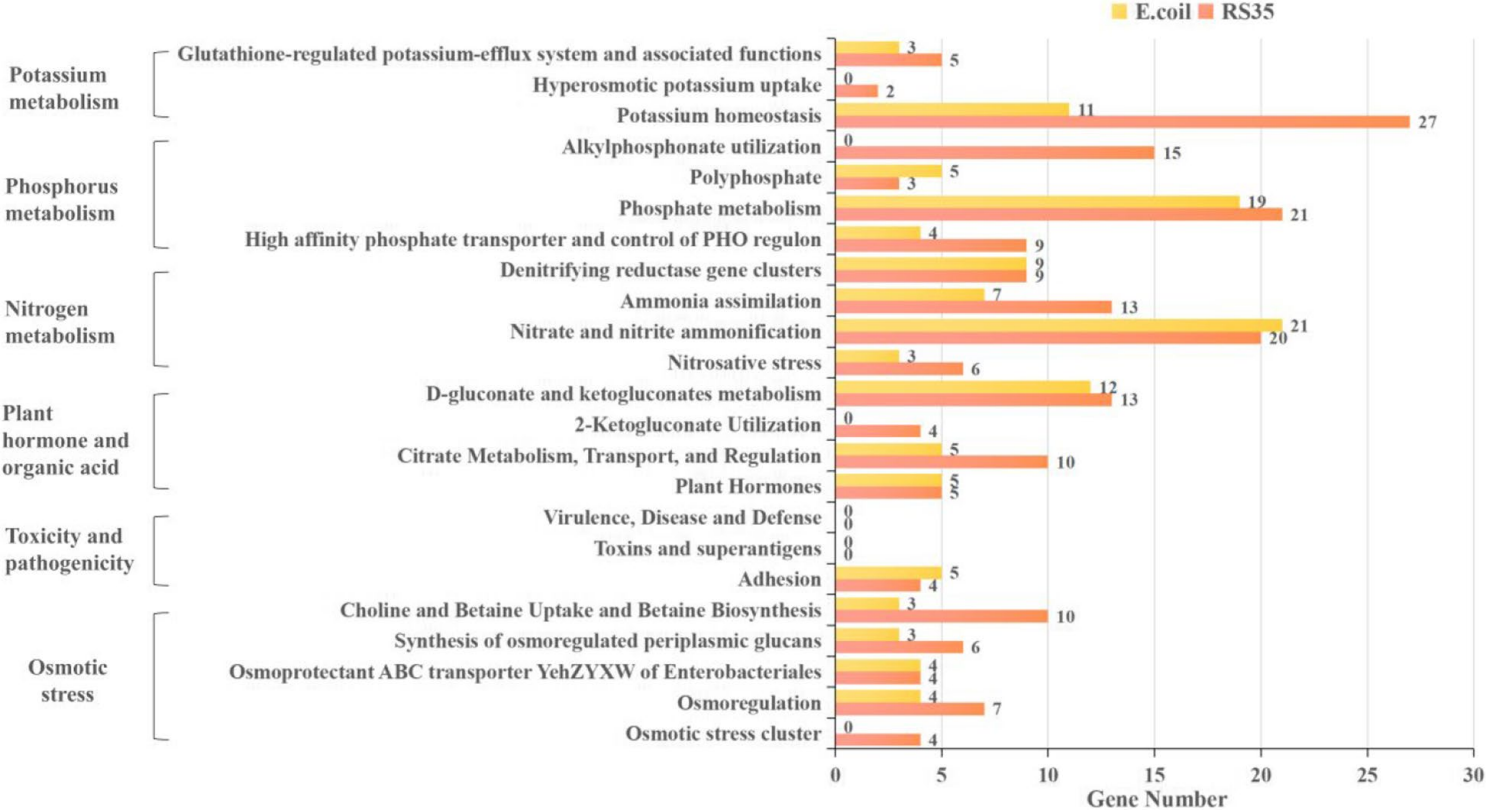
Genome-wide annotation analysis. *E. cloacae* Rs-35 number of annotated genes for osmotic stress, ion uptake, phytohormones, organic acids, and toxic substances-related metabolic pathways in response to the saline environment (take Escherichia coli as control)

The whole genome of *E. cloacae* Rs-35 (GenBank accession no. PRJNA839536) is sequenced and assembled by using third-generation sequencing technology. The total length of the assembled complete genome is 5112849 bp, and the GC content is 54.5%. After analysis and prediction, a total of 4657 coding genes and 111 RNAs were found. The *E. cloacae* Rs-35 genome was annotated by Rapid Annotation Using Subsystem Technology (RAST) online, and the functional gene of the strain was annotated by the comparison library. A total of 4001 annotated genes are associated with 27 subsystems, including 183 related genes annotated by the stress-response-related pathway system. It is noteworthy that the genome of *E. cloacae* Rs-35 strain has multiple genes related to plant growth promotion characteristics (take *Escherichia coli* as control) (Fig. 5). In response to osmotic stress-related pathways in saline and alkaline environments, *E. cloacae* Rs-35 has annotated a total of 31 related genes. In terms of plant hormone metabolism-related pathways, *E. cloacae* Rs-35 has annotated 38 related genes. In the synthesis of plant hormone IAA, 11 tryptophan synthesis genes have been annotated. Tryptophan provides a precursor for the biosynthesis of IAA, and studies have confirmed that the application of tryptophan can improve the growth and development of plants [25]. At the same time, Blast online comparison of the National Center for Biotechnology Information (NCBI, https://www.ncbi.nlm.nih.gov/) confirmed that the genome of *E. cloacae* Rs-35 strain contains the indole pyruvate decarboxylase gene (*ipdC*) and the ACC deaminase gene (*acdS*). *IpdC* encodes the indole pyruvate decarboxylase, which can decarboxylate indole pyruvate to indole acetaldehyde. This reaction is the rate-limiting step of the indole pyruvate pathway-IAA biosynthesis pathway [26, 27]. ACC deaminase is an intracellular enzyme that inhibits ethylene biosynthesis. It can promote plant growth and metabolism by reducing ethylene content [28]. The phylogenetic analysis of the *E. cloacae* Rs-35 strain based on the *acdS* found that the *E. cloacae* Rs-35 strain has the highest similarity with *E. cloacae* SBP8. In terms of ion absorption, 130 genes were annotated in N, P, and K metabolic pathways. In terms of phosphorus dissolving and organic acid metabolism, NCBI's Blast comparison found that the genome of *E. cloacae* Rs-35 strain contains a phosphatase gene (*aphA*), which plays an important role in removing the phosphate group of the substrate in the process of gluconic acid (GA) synthesis. GA is one of the main organic acids of most bacteria, and it plays an important role in the dissolution of inorganic phosphate [29]. At the same time, research shows that 2-keto gluconic acid has a higher phosphorus dissolution efficiency than gluconic acid [30]. The production of 2-keto gluconic acid was found in the metabolic pathway of gluconic acid, malic acid, and citric acid in *E. cloacae* Rs-35, which further shows that it can dissolve phosphorus. Finally, in terms of virulence, pathogenicity, and other pathways, no relevant genes have been annotated, indicating that *E. cloacae* Rs-35 is a non-pathogenic and relatively safe Enterobacterium. In conclusion, the whole genome annotation results show that *E. cloacae* Rs-35 have multiple metabolic pathways related to salt solubilization and growth promotion, further confirming the correctness of the above ion absorption measurement and endogenous hormone experiment.

#### Analysis of the salt relief and growth promotion-related functions of *E. cloacae* Rs-35 based on multi-omics techniques

**Fig. 6:**
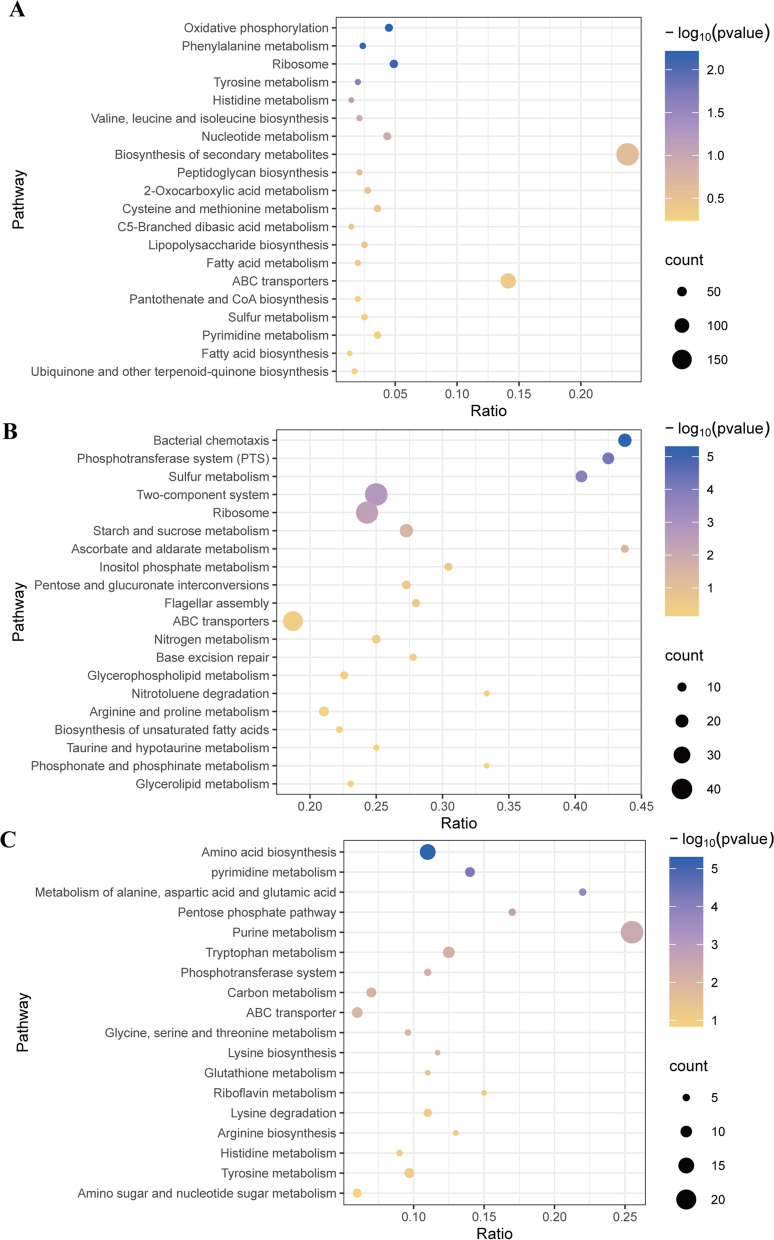
Bubble diagram analysis of the differentially expressed substances on the transcriptome, proteome, and metabolome of *E. cloacae* Rs-35 strain under two conditions. **A** Bubble diagram analysis of differentially expressed genes. **B** Bubble diagram analysis of differentially expressed proteins. **C** Bubble diagram analysis of differential metabolites. Using padj (multiple hypothesis test corrected *p*-value) < 0.05 as the threshold of significant enrichment (*n* = 3)

The *E. cloacae* Rs-35 strain cultured under normal conditions (PH = 7.0, NaCl = 10 g/L) and high saline-alkaline conditions-maximum salt and base tolerance of *E. cloacae* Rs-35 (PH = 10.0, NaCl = 60 g/L) were analyzed by transcriptome, proteome, and metabolomics, and the annotated differentially expressed genes, differentially expressed proteins and differentially expressed metabolites were annotated by Kyoto Encyclopedia of Genes and Genomes (KEGG, https://www.kegg.jp/) function. Through the classification and functional annotation of the database, the main biochemical metabolic pathway and signal transduction pathway of the differentially expressed substances of *E. cloacae* Rs-35 strain under saline-alkali stress were determined. Using padj (multiple hypothesis test corrected *p*-value) < 0.05 as the threshold of significant enrichment, a bubble plot is drawn, the horizontal coordinate is the ratio of the number of differences to the total number of differences, the larger the value indicates the greater the enrichment, the vertical coordinate is the metabolic pathway annotated to, the size of the point represents the number of differences substances annotated to that pathway, the color from blue to yellow represents the significant size of the enrichment, the results are shown in Fig. 6.

At the transcriptional level (Fig. 6A), the differentially expressed genes in the normal and saline groups of *E. cloacae* Rs-35 were significantly enriched to the KEGG pathway having oxidative phosphorylation, phenylalanine metabolism, ribosome, and tyrosine metabolism. At the translational level (Fig. 6B), the differentially expressed proteins of the normal and saline groups of *E. cloacae* Rs-35 were significantly enriched to the KEGG pathway with bacterial chemotaxis, phosphotransferase system, sulfur metabolism, two-component system, ribosome, starch and sucrose metabolism, and ascorbate and aldarate metabolism. At the metabolic level (Fig. 6C), the differential metabolites of the normal and saline groups of *E. cloacae* Rs-35 were significantly enriched to the pathways having amino acid biosynthesis, pyrimidine metabolism, metabolism of alanine, aspartic acid and glutamic acid, pentose phosphate pathway, purine metabolism, tryptophan metabolism, phosphotransferase system, carbon metabolism, ABC transporter, glycine, serine and threonine metabolism, lysine biosynthesis, and glutathione metabolism. From transcription to translation to metabolism, the number of significantly enriched pathways increased, while the number of pathways enriched to differentially expressed substances decreased. The ABC transporter pathway, which is involved in the intracellular transport of Na, was enriched at all three histological levels. Tryptophan metabolism was enriched at the metabolic level, which provides precursors for IAA biosynthesis. Bacterial chemotaxis, phosphotransferase system, and two-component system were significantly enriched at the proteomic level, suggesting that *E. cloacae* Rs-35 may be regulated by its signal transduction to grow well in hypersaline environments. In addition, a large number of metabolic pathways such as amino acid biosynthesis, carbon and glucose synthesis, and oxidative phosphorylation were significantly annotated, and the significant changes of these substances in the cells of *E. cloacae* Rs-35 could indicate its high metabolic capacity in a saline environment. These results further suggest that *E. cloacae* Rs-35 possess the ability to alleviate cotton salt stress and promote cotton growth efficiently when inoculated onto salt-stressed cotton plants.

## Discussion

### Role and mechanism of ion uptake and balance in cotton by *E. cloacae* Rs-35

Salt stress disrupts the homeostasis of intracellular ions and slows down the metabolism of plants. Excessive Na^+^/K^+^ will lead to water shortage, membrane dysfunction, and ion toxicity of plant cells [31]. Therefore, the normal cell function and growth and development of plants need to maintain a low level of Na^+^/K^+^ concentration. Under a salt environment, cotton inevitably absorbs Na^+^, affecting the absorption of other mineral elements, resulting in ion imbalance. It was shown that one of the mechanisms by which PGPR alleviates salt stress in plants is that the extracellular polysaccharides secreted by PGPR strains not only bind cations but also promote the formation of biofilms on the root surface, thereby limiting the internal flow of Na^+^ into the body [32]. The second mechanism is that PGPR strains can regulate the expression of salt tolerance-related genes in host plants [33, 34]. In summary, plants can avoid or reduce salt damage only when low Na^+^ levels are maintained in the plant cytosol, and the ability of cells to reject Na^+^ is mainly controlled by the plasma and vacuole membranes [35]. On the one hand, the Na^+^/H^+^ reverse transporter on the cell membrane will pump Na^+^ out of the cell, and on the other hand through Na^+^/H^+^ reverse transporter on the vacuole membrane will pump Na^+^ that has entered the cytoplasm into the vacuole for storage. Furthermore, H^+^-ATPase on plant membranes is a family of proton pumps driven by ATP hydrolysis, generating a transmembrane proton electrochemical gradient, whose activity can characterize the activity of transporters on the plasma membrane [36]. It is a prerequisite for the nutrient absorption and growth of plant cells [37]. Thus, the higher activity of the membrane H^+^-ATPase is also critical for plant adaptation to salt-stress environments.

This article shows that *E. cloacae* Rs-35 could significantly reduce the absorption of sodium ions by cotton roots and leaves, promote the absorption of potassium and calcium ions, regulate the ratio of K^+^/Na^+^ and Ca^2+^/Na^+^, and maintain the ion balance of cotton plants. The activity of H^+^-ATPase on the plasma membrane and vacuolar membrane of the roots and leaves of cotton plants inoculated with *E. cloacae* Rs-35 changed greatly, indicating that the sensitivity of cotton to salt significantly increased under the effect of *E. cloacae* Rs-35. At the same time, studies showed that several transporters and kinases *kdpD*/*kdpE* were found in the whole genome of the D5A strain isolated from alkaline soil, which can help plants alleviate salt stress [38]. Therefore, the genes related to the reverse transporter of strain *E. cloacae* Rs-35 were investigated, and it was found that *E. cloacae* Rs-35 effectively alleviated the salt stress on cotton by affecting the activity of Na^+^/H^+^ reverse transporter on the vacuolar membrane of cotton leaves. Further, genome-wide annotation and multi-group analysis showed that the genes encoding the K^+^ transport system and Na^+^/H^+^ reverse transport system were found in the *E. cloacae* Rs-35 genome. These genes were used to introduce H^+^ and pump out Na^+^. The discovery of these genes confirmed that *E. cloacae* Rs-35 has the function of salt solubilization and growth promotion in ion absorption at the molecular level.

### Role and mechanism of endogenous hormones in cotton by *E. cloacae* Rs-35

PGPR can affect crops by interfering with plant endogenous hormones or providing exogenous hormones, which are indispensable for plant growth and metabolism. Synthesis of IAA is characteristic of many rhizospheres’ growth-promoting bacteria, *E. cloacae* Rs-35 is no exception. Experiments showed that *E. cloacae* Rs-35 treatment would have an effect on the content of endogenous hormones in cotton under salt stress, with little effect on ABA content and a significant effect on IAA content, so that cotton could maintain a relatively stable IAA content under salt stress. A total of 12 tryptophan (synthetic precursor of IAA) synthesis genes were also found to be annotated in their whole genome by genome-wide annotation and significantly enriched to the tryptophan metabolic pathway at the metabolome level in differentially saline environments. The synthesis of IAA with tryptophan as a precursor can be divided into five categories: indole acetonitrile, tryptophan, tryptophan side chain oxidase, indole-3-pyruvate, and indole acetamide. Bacteria can synthesize IAA in one or more ways. In the indole-3-pyruvate (IpyA) pathway [39], tryptophan is first oxidized and deaminated to form indole-pyruvate, and then indole-pyruvate is oxidized to form indole acetaldehyde through the action of indole-pyruvate decarboxylase and finally oxidized to IAA. The gene *ipdC* was found in the whole genome of *E. cloacae* Rs-35. *IpdC* is the key gene for the synthesis of indole-3-acetic acid via the indole-pyruvate pathway, indicating that the strain *E. cloacae* Rs-35 may synthesize IAA via the IpyA pathway, but it still needs to be further verified by experiments. ACC deaminase can degrade ACC, the precursor of ethylene, to produce ammonia and hydroxybutyric acid, reduce the amount of ethylene in plants, and promote plant growth. McDonnell et al. [40] found that the At1g48420 gene encoding the d-cysteine desulfurizing enzyme has ACC deaminase activity. After comparison, the *acdS* sequence similarity between *E. cloacae* Rs-35 and *E. cloacae* SBP8 is the highest, reaching 98.58%, indicating that *E. cloacae* Rs-35 may have the ability to degrade ethylene.

### Multi-omics sequencing reveals the metabolic mechanism of *E. cloacae* Rs-35 to alleviate salt stress in cotton

The improvement of salinity tolerance in cotton plants by PGPR is a complex regulatory mechanism arising from microbial-plant interactions, and the process needs to be explored in depth from several aspects. It is important to explore the various desalination-promoting metabolic activities of PGPR itself at the molecular level using the emerging multi-omics sequencing technology. In this paper, we elucidate the physiological metabolic mechanisms of salt stress mitigation in cotton by *E. cloacae* Rs-35 from genomics, transcriptomics, proteomics, and metabolomics. It is shown that in addition to the above-mentioned phytohormone metabolism and N, P, and K metabolism, *E. cloacae* Rs-35 can improve the salt tolerance of cotton in terms of osmoregulatory substances, phosphorus dissolved, and organic acid metabolism in response to a saline environment.

Phosphorus in soil exists in the form of insoluble phosphorus, which is difficult to absorb and utilized by plants. Phosphate-dissolving microorganisms can convert phosphorus, which is difficult for plants to absorb and use, into a form that plants can absorb and use [41]. Phosphorus-dissolving mechanism of phosphorus-dissolving bacteria varies with different strains. At present, more research is focused on phosphatase, hydrogen ions, and organic acid secretion [42]. In this paper, *aphA* was identified in the *E. cloacae* Rs-35 strain, which can encode the production of acid phosphatase, and acid phosphatase can hydrolyze phosphate groups from different organic phosphorus substrates for plant absorption and utilization, which plays an important role in the phosphorus dissolution process of the strain. At the same time, the production of 2-keto gluconic acid was found in the metabolic pathway of gluconic acid, malic acid, and citric acid in *E. cloacae* Rs-35, which further showed that it could dissolve phosphorus.

The accumulation of osmoregulation substances such as proline, polysaccharide, and betaine is a sensitive indicator of plant response to saline-alkaline stress. PGPR enhances plant saline-alkaline resistance by secreting these osmoregulation substances [43]. The genes related to osmoregulation substances were screened in the *E. cloacae* Rs-35 strain, and the genes encoding betaine aldehyde dehydrogenase and choline dehydrogenase were found, which played an important role in the formation of betaine. In addition, the genes encoding reaction enzymes *treB*, *treY, treZ*, *treR*, *otsB,* and *ostA* in trehalose synthesis were found in *E. cloacae* Rs-35, which played a key role in trehalose synthesis.

The transcriptomic, proteomic, and metabolomic analyses of the differential saline environment have led to the annotation of several pathways related to desalination and proliferation, starting from the physiological metabolism of *E. cloacae* Rs-35. ABC transporter protein is a metabolic pathway annotated at all three histological levels, which is essential for water transport and ion homeostasis [44]. Intracellular phosphorylation processes contribute to enhanced salt tolerance [45], and *E. cloacae* Rs-35 is annotated to phosphorylation or phosphate metabolism at both the transcriptional and translational levels. Transcriptome analysis has shown that PGPR enhances the metabolic pathways of ascorbic acid, pentose, and glucuronide in cotton [46], and similarly, multiple sugar and organic acid metabolisms were found in the metabolic pathways of *E. cloacae* Rs-35. The significant enrichment of these metabolic pathways in bacterial chemotaxis, phosphotransferase system, and the two-component system suggests that *E. cloacae* Rs-35 can not only regulate its signal transduction to adapt to high salinity environment but also may induce salinity stress tolerance in cotton to promote cotton growth [20].

## Conclusion

The role of PGPR in promoting plant growth and improving plant tolerance has been widely recognized, and inter-root probiotic bacteria have shown an indispensable role in sustainable agricultural development by reducing the heavy use of synthetic fertilizers and pesticides. In this paper, we investigated the mechanism of ion uptake in cotton, measured endogenous hormones (ABA and IAA) in cotton, performed genome-wide annotation and multi-omics differential analysis, and confirmed that *E. cloacae* Rs-35 can promote the growth and improve the salt tolerance of cotton in the saline environment from both cotton plants and PGPR strains. On the one hand, it indicates that *E. cloacae* Rs-35 can promote the ion uptake ability of cotton in a salt environment and stabilize the IAA hormone level of cotton plants; on the other hand, the results of genome-wide annotation and multi-omics analysis explain that *E. cloacae* Rs-35 can help desalination and promote growth from the perspective of genomic information direction and functional annotation of differentially expressed substances. It is inferred that the mechanisms of *E. cloacae* Rs-35 affecting cotton growth under salt stress may be: providing soluble phosphate for plant growth and stabilizing IAA hormone changes in vivo, improving ion uptake in cotton, producing ACC deaminase to reduce ethylene content, stimulating the induction of systemic tolerance mechanisms, regulating multiple desalination and pro-growth related metabolic pathways through plant signal transduction pathways, and finally promoting cotton plant growth under salt stress. The growth of cotton plants under salt stress was ultimately promoted through the regulation of several salinity-promoting metabolic pathways by plant signaling pathways. Finally, the metabolic mechanism study based on the multi-omics level in this paper helps to reveal the mechanism of Gram-negative bacteria such as *Enterobacteriaceae*, *Pseudomonas,* and so on assisting plant salt relief and growth promotion, which can broaden the thinking and perspective within the field of PGPR-plant interactions research, and is expected to provide some new insights for other and subsequent research teams.

## Data Availability

The genome is available in NCBI (GCF_023702375.1). The datasets generated and/or analysed during the current study are available in the Mendeley Data repository, https://doi.org/10.17632/s2rfjht5xk.1.

## Abbreviations

PGPR: Plant growth promoting rhizobacteria
K^+^: Potassium ion
Ca^2+^: Calcium ion
Na^+^: Sodium ion
H^+^: Hydrogen ion
IAA: Indole-3-acetic acid
NCBI: National Center for Biotechnology Information
NA: Nutrient agar
OD: Optical density
N: Nitrogen
P: Phosphorus
K: Potassium
Na: Sodium
Ca: Calcium
Fe: Iron
Mg: Magnesium
EGTA: Ethylene glycol tetra acetic acid
PMSF: Phenylmethyl sulfonyl fluoride
EDTA: Ethylene diamine tetraacetate acid
PVPP: Polyrinopolypyridone
DTT: Dithiothreitol
BSA: Bovine serum albumin
PCR: Polymerase chain reaction
BSA2: N, O-bis-trimethylsilyl acetamide
GC: Gas chromatography
ABA: Abscisic acid
RAST: Rapid Annotation Using Subsystem Technology
LB: Luria–Bertani
NaCl: Sodium chloride
KEGG: Kyoto Encyclopedia of Genes and Genomes
IpyA: Indole-3-pyruvate
